# Genome-Wide Identification of the TCP Gene Family in *Astragalus mongholicus* and Analysis of Its Response Patterns to Abiotic Stress

**DOI:** 10.3390/biology15141134

**Published:** 2026-07-12

**Authors:** Panpan Wang, Xinxin Wang, Meitong Pan, Zhenzhen Li, Lingyang Kong, Wei Ma, Xiubo Liu

**Affiliations:** 1 College of Pharmacy, Heilongjiang University of Chinese Medicine, Harbin 150040, China; 15134532248@163.com (P.W.); a1302196384@126.com (X.W.); meitong_pan@163.com (M.P.); 15036413064@163.com (Z.L.); hljkly970219@163.com (L.K.); 2 College of Jiamusi, Heilongjiang University of Chinese Medicine, Jiamusi 154007, China

**Keywords:** *Astragalus mongholicus*, TCP transcription factors, gene family identification, abiotic stress, expression profiling

## Abstract

*Astragalus mongholicus* is one of the original plant species of the medicinal herb Astragali Radix as recorded in the Chinese Pharmacopoeia, possessing significant medicinal value and being widely utilized worldwide. TCP transcription factors constitute a plant-specific transcription factor superfamily that plays essential regulatory roles in vegetative growth, organ development, and abiotic stress responses. However, the TCP gene family in *A. mongholicus* has not yet been systematically investigated, and its functional characteristics remain largely unknown. In this study, based on genome-wide data, a total of 25 *AmTCP* genes containing complete TCP domains were identified in *A. mongholicus*, and their protein properties, sequence alignment, gene structures, and phylogenetic relationships were systematically characterized using comprehensive bioinformatics tools. Promoter cis-element analysis revealed that the *AmTCP* promoter regions are enriched in cis-elements associated with hormones, light, and abiotic stresses (drought and salinity), suggesting their potential involvement in multiple signaling cascades. Furthermore, transcriptomic profiling combined with qRT-PCR validation demonstrated that *AmTCP* genes exhibit tissue-specific expression patterns and differential responses to salt stress and polyethylene glycol (PEG)-simulated drought stress. Notably, *AmTCP3*, *AmTCP8*, *AmTCP11*, *AmTCP17*, and *AmTCP19* displayed tissue- and time-dependent alterations in expression under stress conditions, with *AmTCP17* and *AmTCP19* showing the most pronounced responsiveness. Collectively, our findings systematically elucidate the fundamental molecular characteristics of TCP transcription factors in *A. mongholicus*, providing a valuable reference for future investigations into the biological functions of this gene family during growth, development, and abiotic stress responses in this medicinal plant.

## Simple Summary

TCP transcription factors are vital plant-specific regulators essential for vegetative growth, organ development, and abiotic stress responses. *Astragalus mongholicus*, a leguminous medicinal plant, possesses significant pharmaceutical value and is widely utilized worldwide. Although the *TCP* gene family has been documented in numerous plant species, it remains largely uncharacterized in *A. mongholicus*. In this study, we not only identified all *TCP* genes in the *A. mongholicus* genome at the genome-wide level but also performed systematic bioinformatics analyses. Concurrently, we investigated the expression patterns of *AmTCP* genes across different tissues of *A. mongholicus* and their responses to salt and drought stresses. These findings will provide a crucial theoretical foundation for further elucidating the functional roles of *AmTCP* genes in plant developmental regulation and environmental adaptation.

## 1. Introduction

The TCP transcription factors constitute a plant-specific transcription factor superfamily, which derives its name from its initially characterized members: TEOSINTE BRANCHED1 (TB1) in *Zea mays*, CYCLOIDEA (CYC) in *Antirrhinum majus*, and PROLIFERATING CELL FACTORS 1/2 (PCF1/2) in *Oryza sativa* [1]. This superfamily is distinguished by a highly conserved non-canonical basic helix-loop-helix domain spanning roughly 59 amino acids, which critically facilitates sequence-specific DNA binding and protein dimerization. Evolutionary divergence and core structural features partition these regulatory proteins into two primary evolutionary lineages: Class I, synonymous with the PCF subfamily, and Class II, which is further partitioned into the CIN and CYC/TB1 clades. Distinctly, the presence of a conserved four-residue gap within the TCP domain differentiates Class I members from their Class II relatives [2]. As central switches governing vegetative expansion, organogenesis, and defense networks, *TCP* factors represent a major focal point in plant biology. Consequently, genome-wide screening strategies have been widely deployed recently to profile this family in numerous therapeutic herbs. For instance, 17 *LjTCP* genes were identified in *Lonicera japonica* [3], 17 *CsTCP* genes in *Cannabis sativa* [4], 23 *ApTCP* genes in *Andrographis paniculata* [5], and as many as 60 *PgTCP* genes in *Panax ginseng* [6]. Collectively, these investigations not only enrich the current knowledge of TCP family diversity across the plant kingdom but also provide a foundational platform for future functional studies. Importantly, they pave the way for deciphering the sophisticated molecular networks through which TCP transcription factors modulate key agronomic traits, including secondary metabolite biosynthesis, quality formation, and stress tolerance in medicinal plants.

Accumulating evidence has unequivocally established TCP transcription factors as pivotal regulators across a broad spectrum of plant life activities. Evidence indicates that *Arabidopsis thaliana AtTCP14* and *AtTCP15* orchestrate seed germination profiles by participating in the gibberellin response network while concurrently altering foliar cellular development and stem internode elongation [7]. Similarly, transgenic expression of *OsPCF7* in rice alters critical agronomic traits, resulting in seedlings with optimized plant height, root architecture, and tiller numbers, as well as superior panicle formation and grain yield components [8]. Mechanistically, *TCP* family members bolster stress adaptation traits by integrating control of osmotic adjustments, signaling pathways, phytohormone sensitivity, and antioxidant defense mechanisms. For instance, overexpression of *ZmTCP42* in maize confers substantially improved drought tolerance [9], whereas *GbTCP5* in sea-island cotton (*Gossypium barbadense*) directly activates downstream genes such as *GbERD7*, thereby significantly augmenting adaptive capacity to both drought and salt stresses [10]. Additionally, these regulatory proteins actively direct metabolic flows that yield therapeutically valuable secondary metabolites, such as phenolic compounds and volatile terpenoids [11]. Taken together, these insights highlight TCP proteins as vital integration nodes that cross-talk between morphogenesis, environmental defense networks, and specialized metabolic circuits.

*Astragalus membranaceus* (Fisch.) Bunge. var. *mongholicus* (Bunge) P.K. Hsiao, a perennial herbaceous species of the genus *Astragalus* within the Fabaceae family, is one of the principal botanical sources of the renowned traditional Chinese medicine known as Huangqi [12]. With a documented medicinal application spanning over two millennia, this species has been hailed as the “chief of tonic herbs” in the *Compendium of Materia Medica* [13]. Current biochemical validation underscores that *A. mongholicus* possesses a diverse repertoire of active principles, notably diverse polysaccharides, saponins, and flavonoids, which contribute to its multifaceted clinical properties. These molecules cooperatively trigger significant pharmacological actions, including immunomodulatory, antineoplastic, hypoglycemic, anti-aging, neuroprotective, memory-enhancing, and anti-atherogenic effects [14]. This species is predominantly distributed across the arid and high-altitude regions of northern China, where it has evolved robust adaptive strategies to endure harsh environmental conditions. Nevertheless, both the vegetative growth and the accumulation of pharmaceutically active constituents in *A. mongholicus* remain susceptible to abiotic stressors, including elevated temperatures, drought, and salinity, which consequently impose constraints on the further improvement of its medicinal quality and commercial value [15]. To date, research on *A. mongholicus* has largely focused on resource assessment, phytochemical extraction, and pharmacological evaluations, whereas molecular genetic investigations remain underrepresented. Crucially, a systematic profiling of these specific transcription factors in *A. mongholicus* has not yet been detailed in published studies. Fortunately, the success of recent high-quality genome-mapping initiatives for this herb provides a pivotal reference dataset, offering rich opportunities for molecular design breeding and the genome-wide isolation of crucial genes governing elite plant phenotypes and metabolite production.

To bridge this knowledge gap, the present study undertook a genome-wide identification and systematic characterization of the TCP transcription factor family in *A. mongholicus* using integrated bioinformatic analyses. Specifically, we comprehensively investigated the sequence attributes, phylogenetic evolution, conserved motifs, and cis-acting regulatory elements of the identified *AmTCP* genes. Additionally, the transcriptional dynamics of these loci were tracked via RT-qPCR to profile their tissue specificity and environmental responsiveness under fluctuating conditions, including salt, drought, and temperature extremes. In short, these findings broaden our perspective on TCP functional divergence in therapeutic legumes. This comprehensive dataset serves as a robust platform for future genetic dissection of *AmTCP* family members, highlighting their potential contributions to physiological adaptation and the generation of bioactive molecules.

## 2. Materials and Methods

### 2.1. Plant Materials and Growth Conditions

*A. mongholicus* seeds purchased from the Anguo market were used as experimental materials and were identified by Ma Wei from Heilongjiang University of Traditional Chinese Medicine. To initiate cultivation, *A. mongholicus* seeds were surface-sterilized with 75% ethanol and placed in a dark environment for germination. The resulting sprouts were then moved into a custom hydroponic setup patented by our team (Chinese Patent Number 201220490816.3). This system was continuously supplied with a modified Hoagland solution purchased from Coolaber (Beijing, China). Seedling development proceeded under strictly controlled environmental parameters for 30 days, maintaining a diurnal thermal cycle of 25 °C during the day and 20 °C at night, with a constant 60% relative humidity and a photoperiod of 16 h of illumination and 8 h of darkness. To perform tissue-specific profiling, different plant organs, including roots, stems, and leaves, were harvested individually from pristine 30-day-old seedlings. For each biological replicate, tissues from 10 uniformly sized seedlings were pooled; a total of 3 independent biological replicates were prepared. Immediately after sampling, tissues were flash-frozen in liquid nitrogen and stored at −80 °C prior to extraction procedures.

For simulated stress experiments, the nutrient delivery system for 30-day-old water-cultured plants was altered by adding 200 mM NaCl for salinity stress or 20% weight/volume PEG 6000 for drought mimicry. Untreated plants grown concurrently in pure Hoagland solution served as references. Sampling of roots, stems, and leaves from the experimental and baseline groups was performed at 0, 24, 48, and 72 h post-initiation. For each unique combination of treatment and time interval, identical tissue fragments from 10 separate seedlings were combined to form one independent biological sample, yielding 3 distinct replicates in total [16,17]. All resulting tissue fragments were immediately submerged in liquid nitrogen and kept at −80 °C.

### 2.2. Genome Wide Extraction and Structural Feature Prediction of AmTCPs

The comprehensive genomic dataset for *A. mongholicus* was extracted from the Genomic Plants Genome Database platform (http://www.gpgenome.com/species/109 (accessed on 2 may 2026)). For the thorough discovery of entire TCP superfamily components, an integrated approach combining homology metrics and hidden Markov model sequence profiling was utilized. Briefly, canonical TCP protein sequences from *A. thaliana* were downloaded from the *A. thaliana* Information Resource (https://www.arabidopsis.org/ (accessed on 2 may 2026)) and employed as queries for local BLASTP searches against the *A. mongholicus* protein database using TBtools [18] with an E-value threshold of 1 × 10^−5^. For complementary validation, an HMM search was executed via the integrated HMMER3 engine. This screening relied heavily on the hidden Markov model for the typical TCP structural feature PF03634 harvested directly from the Pfam platform (http://pfam.xfam.org/ (accessed on 4 may 2026)).

Structural confirmation of the integrated protein candidates was performed using the NCBI Batch Web CD-Search Tool (https://www.ncbi.nlm.nih.gov/Structure/bwrpsb/bwrpsb.cgi (accessed on 7 may 2026)) to ensure complete TCP domains, with defective proteins manually filtered out. Based on their physical distribution on chromosomes, the verified *AmTCP* genes were systematically numbered from *AmTCP1* to *AmTCP25*. Characteristics, including molecular weight (MW), theoretical isoelectric point (pI), and instability index, were determined using ExPASy’s ProtParam tool (https://web.expasy.org/protparam/ (accessed on 7 may 2026)) to map the physicochemical profiles of the deduced AmTCP proteins. Furthermore, their subcellular localizations were forecasted via the WoLF PSORT web server (https://wolfpsort.hgc.jp/ (accessed on 7 may 2026).

### 2.3. Phylogenetic Analysis

Phylogenetic relationships among AmTCP and AtTCP proteins were investigated by first aligning amino acid sequences using the MUSCLE algorithm in MEGA 12.0 [19] under default settings. Subsequently, a Neighbor-Joining (NJ) phylogenetic tree was generated with 1000 bootstrap iterations for branch support evaluation, and the tree was then customized in EvolView (https://evolgenius.info//evolview-v2/login (accessed on 12 may 2026)). Adhering to the standard division of *A. thaliana* TCPs (Class I and Class II), the identified AmTCPs were assigned to specific subfamilies to further examine their sequence properties and evolutionary dynamics.

### 2.4. Structural and Conserved Motif Characterization of Genes

The MEME (https://meme-suite.org/meme/accessed on 12 may 2026)accessed on 14 may 2026) web server [20] was used to identify conserved motifs across the predicted AmTCP protein sequences, with the maximum motif count set to 10 and other settings left at their default values. Simultaneously, genomic and coding (CDS) sequences for each TCP family member were isolated from the genome annotation file. Subsequently, we illustrated the exon-intron architecture and motif positions using TBtools’ Gene Structure View.

### 2.5. Genomic Localization and Syntenic Duplication Analysis

A genome-wide synteny analysis was integrated to clarify the spatial distribution and evolutionary duplication patterns of *AmTCP* genes in *A. mongholicus*. We utilized the MCScanX module inside TBtools to distinguish between tandem and segmental duplication processes. Based on the results, Advanced Circos was employed to visualize both intra- and inter-species syntenic relationships, with *C. sativa*, *Malus domestica*, and *O. sativa* selected as reference species for inter-species analysis. The chromosomal positions of *AmTCP* genes were annotated to reveal their evolutionary conservation.

### 2.6. Promoter Cis-Acting Element Analysis of AmTCP Genes

To analyze cis-acting regulatory elements, the 2000 bp genomic sequences upstream of the translation start codon (ATG) of *AmTCP* genes were extracted as promoter regions. These sequences were subsequently submitted to the PlantCARE database [21] for element prediction under default parameters. The identified cis-acting elements were then visualized using TBtools.

### 2.7. Profiling of AmTCP Expression Patterns

For tissue-specific expression profiling, raw RNA-seq datasets from hydro-cultivated *A. mongholicus* (roots, stems, and leaves) were deposited from the NCBI BioProject repository (accession: PRJNA1064679). Transcript abundance for individual *AmTCP* genes was determined using Fragments Per Kilobase of transcript per Million mapped reads (FPKM). To generate the expression heatmap, these FPKM metrics were row-scaled via Z-score normalization and subsequently rendered through TBtools.

### 2.8. Quantitative Real-Time PCR (qRT-PCR) Validation

Total RNA was isolated from roots, stems, and leaves of 30-day hydroponically grown *A. mongholicus* seedlings subjected to control or stress conditions using a plant RNA extraction kit (Hangzhou Xinjing Biotechnology, Hangzhou, China). First-strand cDNA was synthesized using HiScript III RT SuperMix with gDNA wiper (Vazyme, Nanjing, China), and qRT-PCR was performed using ChamQ Universal SYBR Green Master Mix (Vazym, Nanjing, China) on an AriaMx system. The *18S* gene was employed as an internal control. Three biological and three technical replicates were analyzed for each sample. Relative transcript abundance was determined via the 2^−ΔΔCT^ method [22]. Primers were designed with Primer3web (https://primer3.ut.ee/ (accessed on May 20)) and are presented in Table 1. Data are shown as means ± SD, plotted using GraphPad Prism 8.0.2, and compared using two-tailed Student’s *t*-test (*p* < 0.05).

## 3. Results and Analysis

### 3.1. Identification and Physicochemical Property Analysis of the AmTCP Gene Family

To comprehensively identify *TCP* gene family members in *A. mongholicus*, two complementary strategies involving homologous sequence alignment and HMM searches were integrated. *A. thaliana* TCP protein sequences were used as queries for BLASTP searches with an E-value threshold of 1 × 10^−5^, while HMM searches were performed targeting the conserved TCP domain (PF03634) to obtain initial candidates. Sequences lacking complete TCP domains were subsequently eliminated using the NCBI Batch-CDD tool. Consequently, 25 members harboring intact TCP domains were identified in the *A. mongholicus* genome and sequentially designated AmTCP1-AmTCP25 based on their chromosomal coordinates (Table 2).

Subsequently, the physicochemical properties of these 25 AmTCP proteins were characterized using ExPASy (https://web.expasy.org/protparam (accessed on May 21)). The results showed that the protein lengths ranged from 1–39 (AmTCP15) to 498 aa (AmTCP17), with corresponding molecular weights varying from 15.19 kDa (AmTCP15) to 53.03 kDa (AmTCP17). The theoretical isoelectric points (pIs) spanned from 5.17 (AmTCP15) to 9.82 (AmTCP1). All AmTCP proteins exhibited negative GRAVY values, indicating their hydrophilic nature. Furthermore, subcellular localization prediction indicated that all AmTCP proteins were localized to the nucleus, consistent with the established functional characteristics of TCP transcription factors.

### 3.2. Partition Characteristics of the bHLH Domain in AmTCP Proteins

**Figure 1:**
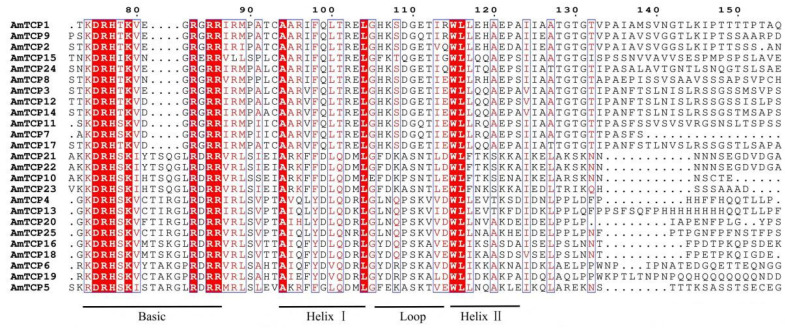
Multiple sequence alignment analysis of the *AmTCP* gene family.

Multiple sequence alignment and sequence logo analysis demonstrated that all AmTCP proteins contained an atypical 59aa bHLH domain. Within this domain, the basic region, Helix I, and Helix II exhibited high conservation. The basic region was particularly enriched in basic amino acids, including R, H, and K. Conserved repeated W and L residues were observed in Helix II, while the A and L sites within Helix I were also highly conserved (Figure 1). Domain segmentation analysis confirmed that all AmTCP proteins shared a conserved four-segment architectural pattern: basic region, Helix I, Loop, and Helix II, and that this canonical scaffold was retained across all tested sequences. Distinct amino acid variations were detected in Helix I, the Loop, and Helix II among different members, suggesting that these variations may underlie the functional divergence of this gene family.

### 3.3. Phylogenetic Analysis of the AmTCP Gene Family

**Figure 2:**
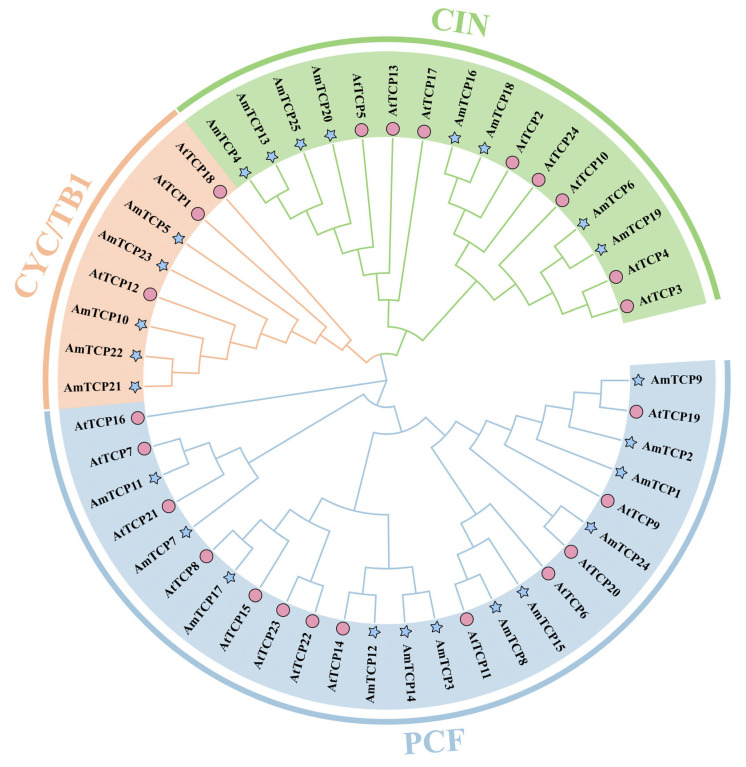
Phylogenetic tree of TCP proteins from *A. mongholicus* and *A. thaliana*. Different subfamilies are highlighted with distinct colored backgrounds. Red circles and blue stars denote TCP proteins from *A. thaliana* and *A. mongholicus*, respectively.

To elucidate the evolutionary dynamics of the TCP family in *A. mongholicus*, a phylogenetic tree was generated using the full-length protein sequences of 25 AmTCP and 26 *A. thaliana* AtTCP members. This reconstruction was performed in MEGA by applying the Neighbor-Joining algorithm, with statistical confidence validated by 1000 bootstrap iterations (Figure 2). The resulting phylogenetic topology clearly divided the 25 AmTCP proteins into two primary subfamilies, Class I and Class II. Specifically, Class I aligned with the PCF clade and contained 12 AmTCP members, whereas Class II was subdivided into two distinct subclades: the CYC/TB1 group, consisting of 5 AmTCPs, and the CIN group, which included 8 AmTCP members.

### 3.4. Motif, Domain, and Gene Structure Analysis of AmTCP Gene Family Members

**Figure 3:**
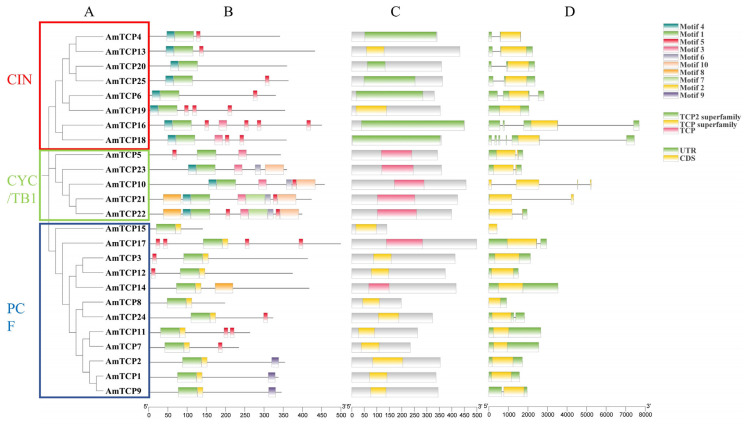
Conserved motif and gene structure analysis of the TCP gene family in *A. mongholicus*. (**A**) Phylogenetic tree constructed based on the full-length protein sequences of 25 AmTCP members using the Neighbor-Joining (NJ) method with 1000 bootstrap replicates. (**B**) Conserved motif distribution diagram, in which 10 different colored boxes represent 10 distinct motifs. (**C**) Conserved domain analysis. (**D**) Gene structure schematic, in which yellow boxes represent exons (CDS), gray lines represent introns, and blue boxes represent untranslated regions (UTRs).

To further elucidate the structural diversity of the *TCP* gene family in *A. mongholicus*, gene structure maps were constructed in TBtools using alignments of coding sequences and full-length genomic sequences. Concurrently, conserved motifs were identified using the MEME online tool to map the annotated TCP domains (Figure 3). The results demonstrated that all 25 *AmTCP* genes contained the canonical TCP domain. Members within the same subfamily exhibited highly similar motif compositions and structural features, whereas pronounced differences were observed between subfamilies (Figure 3A). A total of 10 conserved motifs were identified via the conserved motif analysis (Figure 3B). Specifically, Class I members commonly shared Motifs 1 and 4, whereas Class II members uniquely possessed Motifs 7 and 9. In addition, untranslated regions (UTRs) were identified at the 5′ or 3′ ends of several genes (Figure 3C). Genomic structure analysis revealed that the number of exons in *AmTCP* genes varied from 1 to 6. Notably, Class I members generally had fewer exons, whereas Class II members had a relatively higher number, aligning with the more complex gene structural architectures acquired by Class II members during the evolutionary diversification of the TCP gene family (Figure 3D).

### 3.5. Chromosomal Localization and Gene Duplication Analysis

**Figure 4:**
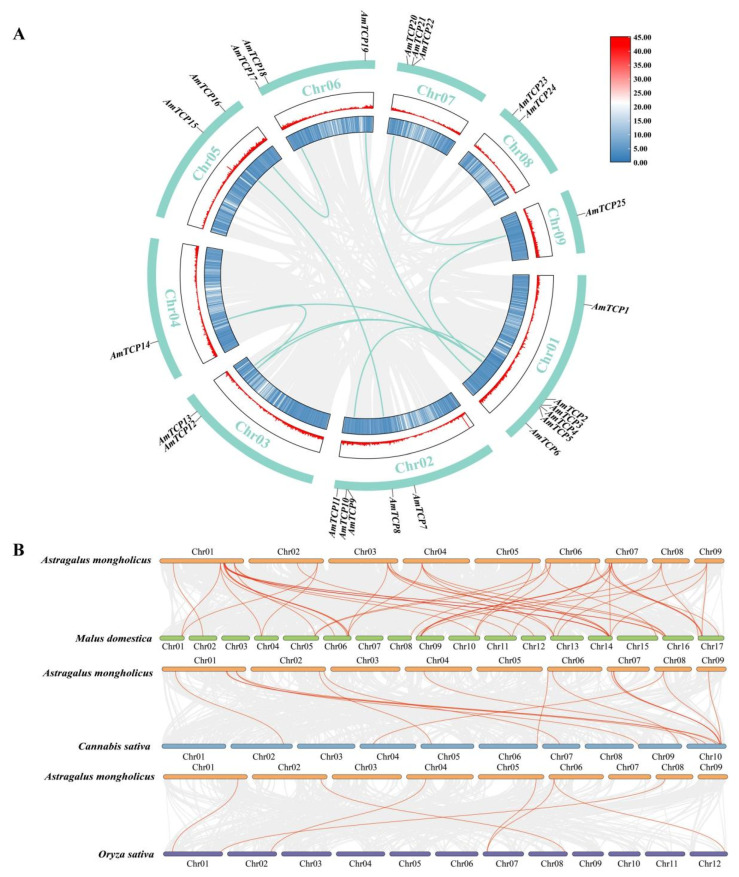
Chromosomal localization and synteny analysis of the *AmTCP* gene family. (**A**) Chromosomal distribution of *AmTCP* genes and intragenomic synteny analysis in *A. mongholicus*. (**B**) Intergenomic synteny analysis between *A. mongholicus* and other representative species.(Red lines represent *AmTCP* genes in homologous pairs. Gray lines symbolize the colinear blocks within *A. mongholicus* and other genomes).

A total of 25 non-redundant *AmTCP* genes were identified within the *A. mongholicus* genome and assigned the identifiers *AmTCP1*-*AmTCP25* according to their physical chromosomal locations. These genes exhibit an irregular distribution pattern across nine chromosomes. Notably, chromosome 1 harbors the most extensive cluster, with six members, while chromosomes 4 and 9 exhibit the sparest distribution, each carrying only one *AmTCP* gene (Figure 4A). Furthermore, inter-species collinearity investigations identified 49, 16, and 7 orthologous *TCP* gene pairs between *A. mongholicus* and *M. domestica*, *C. sativa*, and *O. sativa*, respectively (Figure 4B). These results imply that substantial interspecific duplication, likely the result of whole-genome duplication, played a pivotal role in the expansion of the *AmTCP* family. The higher count of orthologous pairs observed between *A. mongholicus* and dicotyledonous species indicates closer evolutionary divergence and a greater number of conserved ancestral loci than the more distant relationship with the monocot *O. sativa*.

### 3.6. Cis-Acting Element Analysis

**Figure 5:**
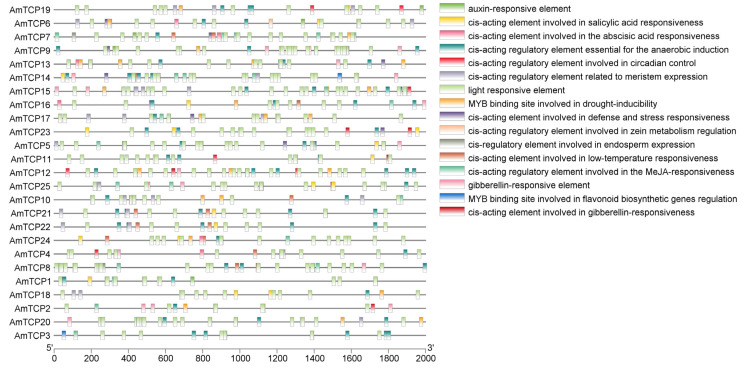
Cis-element analysis of *AmTCP* promoters.

We identified 25 non-redundant *AmTCP* genes in the *A. mongholicus* genome (*AmTCP1-AmTCP25*), which are non-uniformly distributed across nine chromosomes. Notably, chromosome 1 contains the largest gene cluster (six members), whereas chromosomes 4 and 9 host only one gene each (Figure 5).

Collinearity analysis identified 49, 16, and 7 orthologous pairs between *A. mongholicus* and *M. domestica*, *C. sativa*, and *O. sativa*, respectively. The high frequency of interspecies duplications, likely attributable to whole-genome duplication, appears to be a key driver of the expansion of the *AmTCP* family. The abundance of orthologous pairs with dicotyledonous species, compared with monocot *O. sativa*, points to closer evolutionary ties and better preservation of ancestral loci in *A. mongholicus*. Most *AmTCP* gene promoters concurrently harbored multiple distinct types of cis elements, enabling coordinated regulation by hormonal signals, environmental inputs, and light regimes. Notably, even within the same evolutionary clade, *AmTCP* genes exhibited marked differences in the types and numbers of stress- and hormone-responsive elements within their promoter regions. This suggests that this gene family has evolved complex, divergent expression regulatory patterns over time, potentially fulfilling multifaceted roles in stress adaptation and development in *A. mongholicus*.

### 3.7. Analysis of Expression Patterns and Quantitative Real-Time PCR (qRT-PCR) Verification

**Figure 6:**
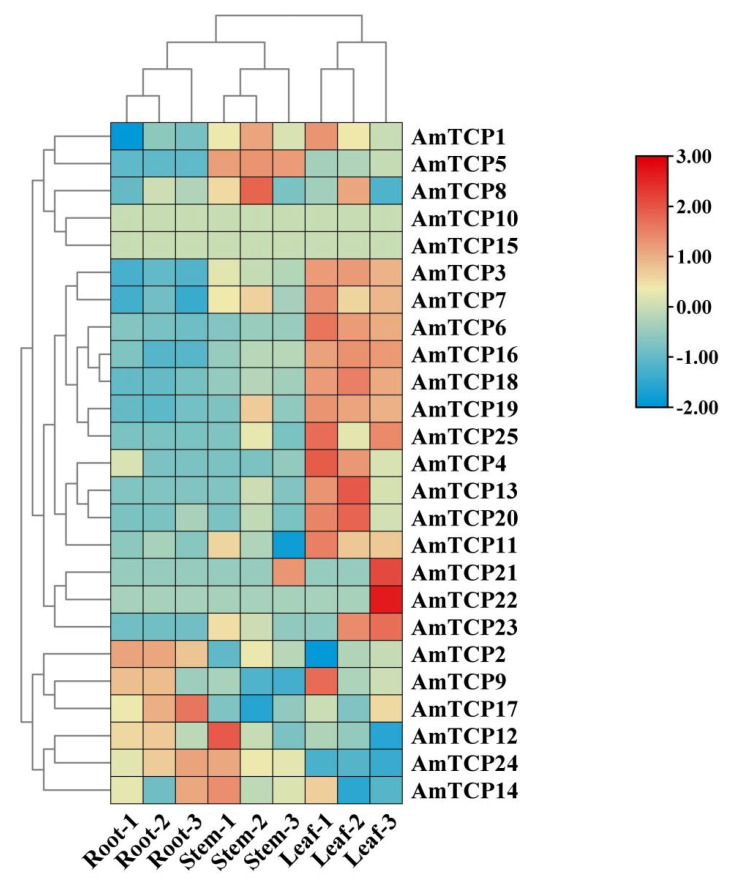
A heatmap was generated to display the expression profiles of *AmTCP* genes in roots, stems, and leaves of *A. mongholicus*. The expression values were log-transformed using Log_2_(FPKM + 1), There were three replicates between groups.

All *AmTCP* genes were expressed in roots, stems, and leaves, indicating their conserved roles in *A. mongholicus* development (Figure 6). However, their expression patterns varied markedly among tissues. Specifically, *AmTCP9*, *AmTCP12*, and *AmTCP17* exhibited root-preferential expression, whereas *AmTCP1*, *AmTCP5*, and AmTCP8 were predominantly expressed in stems. Additionally, *AmTCP4*, *AmTCP13*, *AmTCP20*, *AmTCP21*, *AmTCP22*, and *AmTCP23* displayed leaf-preferential patterns, with *AmTCP22* being exclusively leaf-specific. In contrast, *AmTCP10* and *AmTCP15* maintained moderate and constitutive expression across all three tissues. These findings demonstrate that *AmTCP* members have undergone functional diversification through distinct expression profiles, likely forming a fine-tuned regulatory network to mediate various developmental processes.

**Figure 7:**
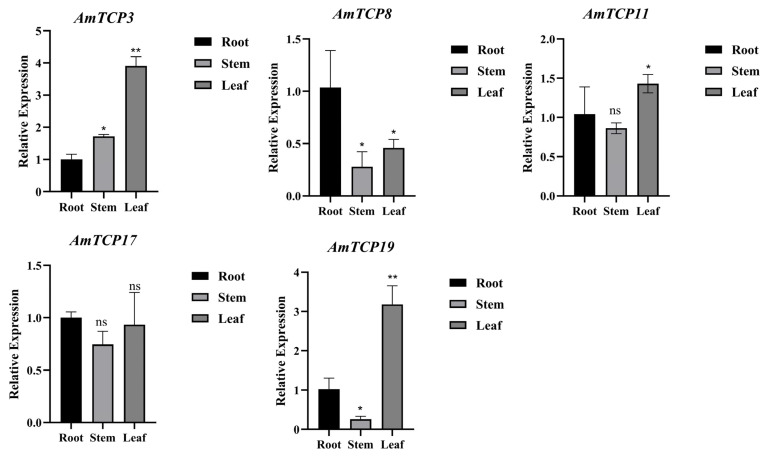
qRT-PCR analysis of *AmTCP* genes in roots, stems, and leaves of *A. mongholicus*. Significance was determined by Student’s *t*-test, with *, and **, indicating significant differences at *p* < 0.05, and *p* < 0.01 respectively, ns indicates no significance.

To verify the accuracy of the transcriptome dataset, we performed RT-qPCR analysis on five representative *AmTCP* genes (*AmTCP3*, *AmTCP8*, *AmTCP11*, *AmTCP17*, and *AmTCP19*) across various tissues (Figure 7). The results demonstrated that all selected genes were expressed in roots, stems, and leaves. Notably, the tissue-specific expression patterns derived from RT-qPCR were highly consistent with the transcriptome data, confirming the robustness and reliability of our RNA-Seq results. Specifically, *AmTCP3*, *AmTCP11*, and *AmTCP19* exhibited peak expression in leaves, whereas *AmTCP8* and *AmTCP17* showed preferential expression in roots. This significant correlation further substantiates the differential tissue expression profiles observed within the *AmTCP* family.

### 3.8. Expression Analysis of Different Tissues in A. mongholicus Under Abiotic Stresses

**Figure 8:**
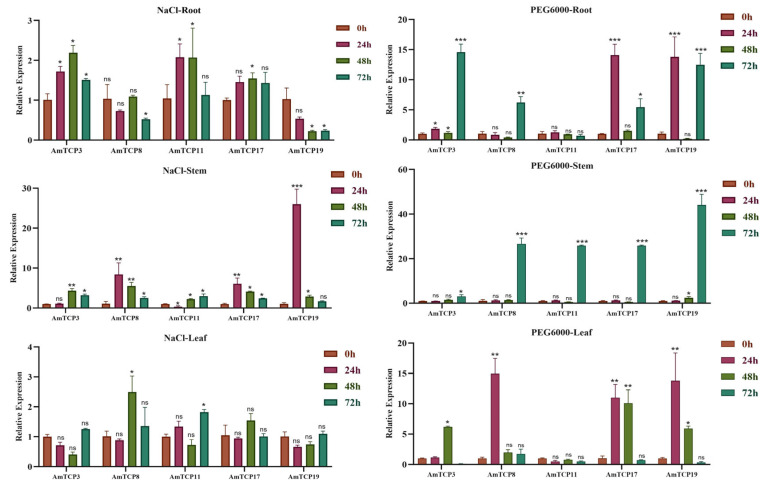
qRT-PCR analysis of *AmTCP* genes under drought and salt stresses. NaCl and PEG were used to simulate salt stress and drought stress, respectively. Statistical significance was determined by *t*-test, where *, ** and *** represent significant differences at *p* < 0.05, *p* < 0.01 and *p* < 0.001, respectively, ns indicates no significance.

To explore the response profiles of *AmTCP* genes to abiotic stresses, RT-qPCR was used to monitor the changes in *AmTCP3*, *AmTCP8*, *AmTCP11*, *AmTCP17*, and *AmTCP19* expression in roots, stems, and leaves under salt and drought treatments. Under salt stress, *AmTCP* genes exhibited distinct tissue-specific response patterns. In roots, the overall induction amplitude was relatively low, primarily characterized by upregulation at the early and middle stages. Specifically, *AmTCP3* and *AmTCP11* were significantly upregulated at 24 to 48 h, and *AmTCP17* was induced at 24 h, whereas *AmTCP8* and *AmTCP19* were generally downregulated. Conversely, stems displayed the most dramatic stress responses. *AmTCP19* was upregulated approximately 30-fold at 24 h, *AmTCP8* and *AmTCP17* were markedly induced at the same time point, and *AmTCP3* peaked at 48 h. The response in leaves was generally weak, with only *AmTCP8* significantly upregulated at 48 h (Figure 8).

Under drought stress, *AmTCP* genes displayed much stronger induction amplitudes and more pronounced temporal dynamics than under salt stress. Roots exhibited a bimodal response pattern, with *AmTCP11*, *AmTCP17*, and *AmTCP19* significantly upregulated at 24 h, followed by a second upregulation peak for *AmTCP3*, *AmTCP8*, and *AmTCP19* at 72 h. Stems showed a typical late-stage response, with all tested genes markedly induced at 72 h, led by *AmTCP19* with a 45-fold increase. Leaf responses primarily occurred during the early stress stage. *AmTCP8*, *AmTCP17*, and *AmTCP19* were significantly upregulated at 24 h, maintained high expression levels at 48 h, and declined to baseline levels at 72 h. Overall, *AmTCP17* and *AmTCP19* were the most highly responsive genes to drought stress. Moreover, *AmTCP19* served as the core responsive component in stems under salt stress, suggesting that these two genes may play vital roles in the abiotic stress regulatory network of *A. mongholicus* (Figure 8).

## 4. Discussion

TCP transcription factors are plant-specific regulatory proteins involved in growth, organ morphogenesis, hormone signaling, and abiotic stress responses, playing essential roles in stress adaptation and metabolic regulation [23]. TCP family size varies considerably across species, with expansion closely linked to genome evolution, whole-genome duplication (WGD), and segmental duplications [24]. *A. mongholicus* is a traditional Chinese medicinal herb whose primary active components are isoflavonoids, yet a systematic study of its TCP family remains lacking. Here, we identified 25 *AmTCP* genes in the *A. mongholicus* genome. This gene count differs from that of other medicinal and model plants, a divergence likely driven by WGD and chromosomal structural variations [25]. Gene duplication drives transcription factor family expansion, with tandem and segmental duplications playing key roles in TCP family amplification and functional divergence [26]. Chromosomal mapping indicates a non-uniform distribution of *AmTCP* genes across nine chromosomes, providing evidence for the significant contribution of segmental duplication to the expansion of this gene family. Furthermore, comparative collinearity analysis demonstrated broad orthologous associations between *A. mongholicus* and relevant dicotyledonous species. In contrast, a notably reduced number of orthologous pairs was detected in comparisons with the monocot rice, attesting to evolutionary divergence occurring after monocot-dicot separation. Conserved motif and gene structure analyses showed high similarity among closely related duplicated genes and members within the same clade, implying functional conservation within subfamilies [27]. Phylogenetic analysis with *A. thaliana* clarified subfamily evolutionary relationships, providing a solid foundation for future functional validation. The *AmTCP* family was classified into Class I and Class II, spanning three canonical subfamilies (PCF, CIN, and CYC/TB1). Notably, the absence of an *AtTCP16* ortholog in *A. mongholicus* aligns with previous findings in Dendrobium [28], suggesting that the *AmTCP* family has developed unique differentiation characteristics and functional specialization during its evolutionary history [29].

Cis-acting element prediction revealed that *AmTCP* promoter regions were enriched with diverse regulatory components, including phytohormone-responsive motifs (abscisic acid, methyl jasmonate, gibberellin, and salicylic acid) and stress-related elements (drought, low temperature, and anaerobic induction). These findings confirm at the transcriptional level that *AmTCP* genes integrate multiple hormonal and environmental signals. Genome-wide analyses across numerous species have similarly demonstrated that *TCP* promoters commonly harbor hormone- and stress-responsive elements, endowing this family with the capacity to cross-talk between multiple signaling pathways [30]. For instance, exogenous MeJA treatment significantly upregulated the expression of *GbTCP6*, *GbTCP11*, and *GbTCP13* in *Ginkgo biloba* leaves [27], while the vast majority of *MeTCP* genes were markedly induced in *Manihot esculenta* under drought stress [31]. Tissue expression analysis showed that *AmTCP* genes were expressed across roots, stems, and leaves, with a ubiquitous expression profile reflecting their fundamental and conserved roles in plant growth. Concurrently, family members displayed distinct tissue-specific differentiation. Some genes were predominantly expressed in roots, others exhibited high specificity in stems or leaves, and a few showed uniform expression across all organs. This spatial divergence indicates that different *AmTCP* genes have undergone functional specialization in specific tissues, forming a fine-tuned molecular network that coordinates *A. mongholicus* development.

TCP transcription factors integrate external environmental inputs and endogenous hormonal signals, participating broadly in abiotic stress responses and regulating tissue development alongside metabolic homeostasis [32]. Because abiotic stress critically affects *A. mongholicus* growth and secondary metabolism, evaluating *AmTCP* expression under salt and drought conditions is key to elucidating these defense mechanisms. Among the 25 *AmTCP* genes, *AmTCP3*, *AmTCP8*, *AmTCP11*, *AmTCP17* and *AmTCP19* with stable expression levels and high representativeness were pre-screened according to transcriptome data. Salt stress and drought stress treatments combined with qRT-PCR were then applied to verify their expression alterations. These genes serve as key candidates of this gene family and provide genetic resources for further functional research on abiotic stress resistance. qRT-PCR validation was performed on these five genes (*AmTCP3*, *AmTCP8*, *AmTCP11*, *AmTCP17,* and *AmTCP19*), confirming the reliability of the transcriptomic data. Under salt stress, tissue-specific induction levels varied markedly among organs. Stems exhibited the highest sensitivity, with all five tested genes significantly upregulated. Notably, *AmTCP19* showed the strongest induction at 24 h, which correlates with the rich abundance of ABA and salt-responsive cis elements in its promoter region. This regulatory pattern is analogous to *FtTCP15* and *FtTCP18* in tartary buckwheat [33] and to *GmTCP22* in soybean [34], supporting a conserved TCP-mediated salt tolerance pathway in plants. Conversely, in roots, *AmTCP3* and *AmTCP11* were upregulated at 24 to 48 h, potentially contributing to root-localized salt signal perception, whereas leaves showed only weak responses. Collectively, these findings indicate that stems serve as the primary regulatory hub for *AmTCP* gene actions during salt stress in *A. mongholicus*.

Under drought stress, *AmTCP* expression patterns differed markedly from those under salt stress, displaying clear stress specificity. In roots, *AmTCP3* peaked at 72 h and served as a key drought-responsive gene. Its promoter harbors an MBS element that directly mediates drought signal activation, consistent with the mechanism reported for cassava *MeTCP* [35]. In stems and leaves, *AmTCP17* and *AmTCP19* were rapidly upregulated at 24 h, enabling early activation of drought defense pathways. Temporal response differentiation was also observed. Specifically, *AmTCP8*, *AmTCP17*, and *AmTCP19* were activated at 24 h to mediate short-term responses, whereas *AmTCP3* and *AmTCP11* peaked at 48 to 72 h to regulate long-term drought adaptation. Previous studies have identified 15 *AmTCP* family genes in the highly salt-tolerant mangrove plant *Avicennia marina* and functionally characterized the orthologous gene *AmTCP8*, providing cross-species comparative references for the findings of this study. In *A. marina*, *AmTCP8* expression is significantly downregulated under salt stress. This gene does not participate in cellular sodium ion transport or the regulation of Na^+^/K^+^ homeostasis. Instead, it directly binds to and activates *AmLOX3*, while simultaneously suppressing the activity of the endogenous ROS scavenging system, leading to substantial accumulation of reactive oxygen species (ROS), and ultimately negatively regulating the salt tolerance of *A. marina* [36].

Under salt stress, the stem of *A*. *mongholicus* serves as the primary responsive organ for *AmTCP* genes. The stem mitigates the damage of salt ions to the leaf photosynthetic system through sodium ion compartmentalization, phloem circulation, and long-distance signal transduction across tissues. Meanwhile, the high expression of *AmTCP* in the stem can regulate the balance between plant growth and stress tolerance, enhancing adaptability to salt stress by inhibiting elongation and promoting lignification. Drought stress can induce stronger and more temporally dynamic expression fluctuations of *AmTCP* genes, primarily attributed to the massive ABA signaling induced by drought that activates upstream ABRE elements in the gene promoters, coupled with circadian rhythm regulation of stomatal metabolism and MAPK cascade signal amplification, collectively driving their dynamic expression. *AmTCP17* and *AmTCP19* are highly sensitive to drought, and *AmTCP19* plays a core role in salt stress response, which is closely associated with the enrichment of numerous stress-related cis-elements in their promoters, flexible phosphorylation modification properties, and their central regulatory status at the transcriptional hierarchy, rendering them as key genes in the abiotic stress response of *A. mongholicus*. Currently, direct evidence regarding the regulation of plant secondary metabolism by TCP transcription factors remains relatively limited, mainly focusing on the effects of the *A. thaliana* TCP family on anthocyanin biosynthesis and the regulation of terpenoid metabolism by TCPs in certain crop species. For *A*. *mongholicus*, an important medicinal plant, this study, for the first time, reveals the tissue-specific and stress-responsive expression patterns of *AmTCP* genes, providing preliminary transcriptomic clues for further exploring whether they participate in the flavonoid and saponin biosynthetic pathways. However, as the current experimental design does not include parallel detection of metabolite contents, the direct causal relationship between *AmTCP* genes and the bioactive components of *A. mongholicus* remains to be validated by subsequent experiments, which will constitute a key research focus of our group in the future.

## 5. Conclusions

In this study, 25 *AmTCP* gene family members were identified from the whole genome data of *A*. *mongholicus.* The TCP family is highly evolutionarily conserved, with segmental duplication playing a dominant role in its expansion. *AmTCP* genes exhibit widespread yet tissue-specific expression patterns, and their transcript levels are significantly modulated by salt and drought stresses, with *AmTCP17* and *AmTCP19* identified as central stress-responsive candidates. These findings systematically characterize the *AmTCP* family and provide valuable candidate genes and theoretical foundations for future studies. Future research should focus on the in vivo functional validation of *AmTCP17* and *AmTCP19*, elucidation of their upstream and downstream signaling networks that regulate abiotic stress tolerance and bioactive compound accumulation, and genetic improvement of stress-resistant, high-quality *A. mongholicus* germplasm through genetic engineering approaches.

## Figures and Tables

**Table 1:** Primers for qRT-PCR.

Primer Name	Forward Primer (5′-3′)	Reverse Primer (5′-3′)
*qAmTCP3*	AAGCCAGCACCAAAACGAAC	TTTCGCCGTCAGATTTGTGG
*qAmTCP8*	AGTTCCTTGCCATGTCCAAC	AACCGCTGCATACTCCATTC
*qAmTCP11*	ATGTCCAACTCCGACGGAACC	TCAACGATGCTCGTCATCTCTTC
*qAmTCP17*	ATTGGGCCACAAATCTGACG	TTGAGAAATTCGCGGGGATC
*qAmTCP19*	TGAAGGTGGTCACATTGTGC	AGAATTCAATGGCGGTGTGG
*18s*	TGCAGAATCCCGTGAACCATC	AGGCATCGGGCAACGATATG

**Table 2:** Sequence feature analysis of the *AmTCP* gene family.

Gene ID	Gene Name	Acid	Molecular Weight	PI	GRAVY	Subcellular Localization
Am01G008970.1	*AmTCP1*	336	36,314.35	9.82	−0.372	nucleus
Am01G025450.1	*AmTCP2*	353	37,435.68	5.91	−0.461	nucleus
Am01G028180.1	*AmTCP3*	412	44,186.62	7.97	−0.723	nucleus
Am01G028880.1	*AmTCP4*	340	38,108.24	6.90	−0.742	nucleus
Am01G029480.1	*AmTCP5*	342	39,297.38	9.16	−1.099	nucleus
Am01G037130.1	*AmTCP6*	329	35,679.95	6.21	−0.681	nucleus
Am02G012260.1	*AmTCP7*	233	25,136.06	9.10	−0.570	nucleus
Am02G018070.1	*AmTCP8*	197	20,981.67	8.44	−0.339	nucleus
Am02G034670.1	*AmTCP9*	344	36,115.19	5.46	−0.357	nucleus
Am02G035150.1	*AmTCP10*	456	52,100.81	6.01	−0.714	nucleus
Am02G039290.1	*AmTCP11*	262	27,949.14	9.56	−0.485	nucleus
Am03G032740.1	*AmTCP12*	373	40,823.70	6.90	−0.784	nucleus
Am03G033630.1	*AmTCP13*	431	47,991.85	7.42	−0.747	nucleus
Am04G014010.1	*AmTCP14*	416	43,842.14	6.90	−0.652	nucleus
Am05G018170.1	*AmTCP15*	139	15,188.25	5.17	−0.275	nucleus
Am05G031460.1	*AmTCP16*	449	49,701.16	7.19	−0.981	nucleus
Am06G000450.1	*AmTCP17*	498	53,027.12	6.18	−0.903	nucleus
Am06G005790.1	*AmTCP18*	357	40,149.90	8.56	−1.043	nucleus
Am06G022640.1	*AmTCP19*	353	38,552.31	5.80	−0.747	nucleus
Am07G003320.1	*AmTCP20*	358	39,872.53	7.76	−0.688	nucleus
Am07G004380.1	*AmTCP21*	422	47,612.48	9.07	−0.768	nucleus
Am07G004610.1	*AmTCP22*	398	45,197.42	9.12	−0.920	nucleus
Am08G002190.1	*AmTCP23*	358	40,185.52	8.26	−0.900	nucleus
Am08G003250.1	*AmTCP24*	322	35,468.39	9.62	−0.730	nucleus
Am09G007870.1	*AmTCP25*	362	40,585.22	8.87	−0.735	nucleus

## Data Availability

The transcriptome data were deposited at the NCBI database under accession number PRJNA1064679.
